# Supplementary material for: Polygenic adaptation on height is overestimated due to uncorrected stratification in genome-wide association studies
Source: eLife. 2019 Mar 21;8:e39702. doi: 10.7554/eLife.39702 (PMC6428571; doi:10.7554/eLife.39702)
Supplement: Supplementary file 3. [file elife-39702-supp3.docx]

**Characterization of stratification effects in GIANT and UK Biobank**

To better understand how stratification influences the differences observed between GIANT and UK Biobank, we grouped SNPs by their P value in GIANT and by their P value in UK Biobank (**Figure 1-figure supplement 1**). First, we observe that SNPs with low GIANT P values, but not SNPs with low UK Biobank P values, show greater differences in estimated effect size (**Figure 1-figure supplement 1a**). However, the relative difference in beta values decreases for lower P values, and the correlation among betas approaches one at the most significant SNPs (**Figure 1-figure supplement 1b,c**). In GIANT, more significant SNPs exhibit a greater correlation between effect estimates and GBR-TSI allele frequency differences, while this is not observed in the UK Biobank (**Figure 1-figure supplement 1d**). Consequently, the difference in UK Biobank and GIANT effect size estimates is more correlated to GBR-TSI allele frequency differences at more significant SNPs (**Figure 1-figure supplement 1e**). This suggests that while stratification effects are larger at more significant SNPs, the magnitude of stratification-independent effects is even larger, which may be why polygenic score results converge when using only the most significant SNPs.

Next, we investigated how P value inflation as measured by $\lambda_{GC}$is influenced by stratification, by grouping SNPs into deciles based on their GBR-TSI allele frequency difference (**Figure 2-figure supplement 3**). To guard against the effect of observing lower P values at more differentiated SNPs simply because those SNPs are more common on average, we restrict this analysis to SNPs with mean MAF > 20%. We find that $\lambda_{GC}$is not much increased for SNPs that are more differentiated between populations. However, in the presence of stratification, there is a large difference between $\lambda_{GC}$of height increasing alleles and $\lambda_{GC}$of height decreasing alleles (**Figure 2-figure supplement 3b**). Similarly, there are large effects on the frequency with which a SNP is estimated to be height increasing or height decreasing. In GIANT, SNPs in the highest decile of GBR-TSI allele frequency differences are 52% more often estimated to be height increasing than height decreasing, while these rates are close to even in the UK Biobank (**Figure 2-figure supplement 3a**).
